# A Revised Hilbert–Huang Transform and Its Application to Fault Diagnosis in a Rotor System

**DOI:** 10.3390/s18124329

**Published:** 2018-12-07

**Authors:** Hongjun Wang, Yongjian Ji

**Affiliations:** 1 School of Mechanical and Electrical Engineering, Beijing Information Science & Technology University, Haidian, Qinghe Xiaoying Donglu No. 12, Beijing 100192, China; jiyongjian@bit.edu.cn; 2 Key Laboratory of Modern Measurement and Control Technology, Ministry of Education, BISTU, Haidian, Qinghe Xiaoying Donglu No. 12, Beijing 100192, China; 3 School of Mechanical Engineering, Beijing Institute of Technology, No. 5 South Zhongguancun Street, Haidian District, Beijing 100081, China

**Keywords:** Hilbert–Huang transform, empirical mode decomposition, end effects, mode mixing, rotor system, fault diagnosis

## Abstract

As a classical method to deal with nonlinear and nonstationary signals, the Hilbert–Huang transform (HHT) is widely used in various fields. In order to overcome the drawbacks of the Hilbert–Huang transform (such as end effects and mode mixing) during the process of empirical mode decomposition (EMD), a revised Hilbert–Huang transform is proposed in this article. A method called local linear extrapolation is introduced to suppress end effects, and the combination of adding a high-frequency sinusoidal signal to, and embedding a decorrelation operator in, the process of EMD is introduced to eliminate mode mixing. In addition, the correlation coefficients between the analyzed signal and the intrinsic mode functions (IMFs) are introduced to eliminate the undesired IMFs. Simulation results show that the improved HHT can effectively suppress end effects and mode mixing. To verify the effectiveness of the new HHT method with respect to fault diagnosis, the revised HHT is applied to analyze the vibration displacement signals in a rotor system collected under normal, rubbing, and misalignment conditions. The simulation and experimental results indicate that the revised HHT method is more reliable than the original with respect to fault diagnosis in a rotor system.

## 1. Introduction

Online monitoring is an effective method to master the running state of machine tools in industrial production. A nonlinear and nonstationary signal is ubiquitous with respect to online monitoring, and there is no doubt that the analysis of this kind of signal is important and difficult [1]. There are several methods for analyzing nonlinear and nonstationary signals, such as the windowed Fourier transform [2], the wavelet transform [3], and the Wigner–Ville distribution [4]. However, almost all of the methods above have their own limitations. For example, wavelet analysis has the advantage of detecting the fast and frequent fluctuations of harmonics, but it is sensitive to noise [5]. Generally, for the wavelet transform, it is important to choose the appropriate wavelet basis function, which also brings about difficulties for signal analysis. The windowed Fourier transform is based on traditional Fourier analysis, so there still remain challenges for processing nonlinear and nonstationary signals.

Huang et al. [6] developed a method for nonlinear and nonstationary signals, which is called the Hilbert–Huang transform (HHT). This method consists of two parts: the empirical mode decomposition (EMD) and the Hilbert spectral analysis (HSA). The Hilbert–Huang transform has a number of advantages over the traditional linear method in analyzing nonlinear and nonstationary signals, since it is highly adaptive in processing a signal. This method has been applied in many scientific fields, such as ocean engineering [7,8,9], business [10], mechanical engineering [11,12,13,14,15,16,17], and other scientific studies [18,19] due to its outstanding performance in signal analysis.

Although HHT is a promising approach to signal processing, it still has some drawbacks, such as end effects and mode mixing. The upper and lower envelopes are obtained by cubic spline interpolation during the process of EMD. Since the local maximum and the local minimum cannot be determined at the starting point and endpoint of the data, serious problems of spline fitting will occur near the ends, where the cubic spline fitting can have large swings, which are called end effects or end swings. The end swings can eventually propagate inward and corrupt the entire data span, especially in the low-frequency components [6]. Rilling [20] did some research on improving EMD. The main methods for suppressing end effects can be divided into four categories: adding window function methods, wave continuation methods, data prediction continuation methods, and extreme continuation methods. The core process of the adding window function methods is adding a symmetric window function, such as a hamming window, a hanning window, or a cosine window, to the data before introducing the HHT operation. The main methods of the wave continuation methods include even prolongation, mirror extending, and periodic extension. Data series forecasting based on a neural network, a support vector regression machine, or an autoregressive moving average model [21] is the typical method of the data prediction continuation methods. Envelope extremum continuation is the main method of the extreme continuation methods. Researchers have proposed numerous methods based on the above methods to eliminate end effects. Qi et al. [22] proposed a novel boundary-processing method based on a cosine window and applied it to diagnose rubbing faults in rotor systems. Zhang et al. [23] applied mirror extending to control end swings. Zhao [24] proposed an improved mirror extending method to optimize the envelopes. Liu et al. [25] proposed a method based on a polynomial fitting algorithm to eliminate end effects. However, there will be a morbid matrix when using this method. Afterwards, Zhu [26] put forward an orthogonal polynomial fitting algorithm to deal with the problem of end swings. Cheng et al. [27] used support vector regression machines to predict the signal before it is decomposed by the EMD method. Yang et al. [28] introduced linear extrapolation to eliminate end swings.

These methods, to a certain extent, solve the problem of end effects, but they are not perfect and they have their own limitations. Although adding a window function can restrain end effects, the original data series also is changed. As a consequence, the intrinsic mode functions (IMFs) that are obtained from EMD cannot express the real component of the original signal, so it is difficult for the Hilbert spectrum to reflect the characteristics of the signal accurately. The wave continuation method requires a signal that has good symmetry, and it is not effective when processing a small amount of data. Neural networks and the autoregressive moving average model are not suitable for forecasting nonlinear and nonstationary signals. A support vector regression machine takes too much time due to the required amount of training data and the calculated quantity of data. In addition, the forecast error will gradually enlarge as the number of prediction steps increases. The extreme continuation method has poor adaptivity, and its effect is not always ideal.

Mode mixing, which is defined as either a single IMF consisting of signals of widely disparate scales or a signal of a similar scale residing in different IMF components, could not only cause serious aliasing in the time-frequency distribution, but could also make the individual IMF lose its physical meaning. Another side effect of mode mixing is the lack of physical uniqueness [29].

The reasons for mode mixing are mainly twofold: (1) the frequencies of the composite components in a mixed signal are similar to each other; and (2) the variation in extremum series. In order to solve the problem of mode mixing, various methods have been proposed by specialists and scholars. Huang et al. [30] were the first to raise the problem of mode mixing, and proposed a solution method that is based on the various adjustable stopping criteria in the sifting processes of the EMD. However, we need to understand the basic characteristics of the signal in advance before using this method. Soon afterwards, Huang et al. [31] applied ensemble empirical mode decomposition (EEMD) to solve the problem of mode mixing. EEMD can eliminate mode mixing by decomposing the mixed signal that contains discontinuous signals. However, it is time-consuming due to the repeated use of EMD. Deering et al. [32] used a masking signal to improve the decomposition effect of EMD. This method effectively separates components that are similar in frequency. Yeh and Shi [33] expanded the masking signal method into general usage. In Yeh and Shi’s research, the masking phase-amplitude coupling (MPAC) method was applied to quantify physiological interactions between high- and low-frequency bands. Tang et al. [34] applied independent component analysis (ICA) to eliminate mode mixing. The ICA method can improve the orthogonality of each IMF; however, it has the limitations of amplitude and sequence uncertainty. At the same time, Tang [35] proposed a new method to eliminate mode mixing based on a revised version of blind source separation. In order to improve the orthogonality of IMFs, Xiao et al. [36] embedded a decorrelation operator into the EMD process. Embedding a decorrelation operator into the EMD process can restrain mode mixing in low-frequency ratio signals; however, it cannot be used to decompose a signal that is mixed with a discontinuous high frequency signal. Hu et al. [37] eliminated mode mixing by an auxiliary signal to the original signal; this method is effective for discontinuous high frequency signals, but not for low-frequency ratio signals.

In order to overcome the limitations of end effects and mode mixing simultaneously, a revised Hilbert–Huang transform is proposed in this article. A new method called local linear extrapolation is introduced to suppress end effects. The advantage of this method is that it can determine the extremum of an endpoint according to the development trend of both ends without extending or predicting the data. The structure of the original data will not be changed with this method, so more original information can be retained. The combination of adding a high-frequency sinusoidal signal to, and embedding a decorrelation operator in, the process of EMD is introduced to eliminate mode mixing. This new method has good performance in eliminating mode mixing even if the multicomponent signal is mixed with high-frequency discontinuous signals and low-frequency ratio signals. In addition, this new method consumes little calculation time.

This paper is organized as follows. The HHT method is reviewed in Section 2. Section 3 presents the revised HHT method. In addition, the question of how to determine the frequency and amplitude of the auxiliary signal is discussed in detail. An experiment on fault diagnosis in a rotor system by the revised HHT is stated in Section 4. Finally, the conclusion is provided in Section 5.

## 2. A Review of the Hilbert–Huang Transform

The Hilbert–Huang transform was proposed by Norden E. Huang, and it consists of two parts: EMD and the Hilbert spectral analysis. Firstly, a data series is decomposed into a series of intrinsic mode functions by the EMD method. Secondly, with the Hilbert transform, the intrinsic mode functions yield instantaneous frequencies as functions of time that give sharp identifications of embedded structures. Finally, an energy-frequency-time distribution can be obtained. In addition, we can also obtain the marginal spectrum of the data series.

EMD is the core method of the Hilbert–Huang transform. According to EMD, the original mixed data can be decomposed into a series of intrinsic mode function (IMF) components. An IMF should satisfy two conditions [6]: in the whole data set, the number of extrema and the number of zero crossings must either equal, or differ at most by, one; at any point, the mean value of the envelope defined by the local maxima and the envelope defined by the local minima must be zero.

Once we have obtained the IMFs according to EMD, then the Hilbert spectral analysis, which is based on the IMFs and the Hilbert transform, can be obtained. Firstly, ${\hat{c}}_{j}(t)$ is calculated by the Hilbert transform, as shown in Equation (1).

(1) $${\hat{c}}_{j}(t)=\frac{1}{\pi }\cdot P\int_{-\infty }^{\infty }\frac{c_{j}(\tau )}{t-\tau }d\tau$$

where *P* indicates the Cauchy principal value. With this definition, $c_{j}(t)$ and ${\hat{c}}_{j}(t)$ form a complex conjugate pair, so we can have an analytic signal, $z_{j}(t)$, as

(2) $$z_{j}(t)=c_{j}(t)+i{\hat{c}}_{j}(t)=a_{j}(t){\cdot e}^{i\theta_{j}(t)}$$

where

(3) $$a_{j}(t)={\left[{c_{j}}^{2}(t)+{{\hat{c}}_{j}}^{2}(t)\right]}^{1/2},\theta_{j}(t)=\arctan\left(\frac{{\hat{c}}_{j}(t)}{c_{j}(t)}\right)$$

where $a_{j}(t)$ is the instantaneous amplitude of $c_{j}(t)$, which can reflect that the energy of $c_{j}(t)$ varies with time, and $\theta_{j}(t)$ is the instantaneous phase of $c_{j}(t)$. The phase is readily obtained, and the instantaneous frequency of each IMF can be defined by the derivative of the phase, as shown in Equation (4)

(4) $$\omega_{j}(t)=\frac{d\theta_{j}(t)}{dt}.$$

We can express the data X(t) in the following form, which does not contain the residue $r_{n}$

(5) X(t)=Re∑j=1naj(t)⋅ei∫ωj(t)dt.

H(ω,t) is defined as the Hilbert amplitude spectrum

(6) H(ω,t)=Re∑j=1naj(t)⋅ei∫ωj(t)dt.

With the Hilbert amplitude spectrum defined, we can also define the marginal spectrum as

(7) $$h(\omega )=\int_{0}^{T}H(\omega ,t)dt.$$

## 3. A Revised Hilbert–Huang Transform (HHT)

Although the Hilbert–Huang transform (HHT) has prospects for widespread application in processing nonlinear and nonstationary signals, the problems of end effects and mode mixing unavoidably influence the accuracy of the final analysis results. In order to obtain a more accurate analysis result, a revised HHT method is proposed. Since the process of EMD is a vital link in HHT, this revised method mainly solves the problems of end effects and mode mixing during the process of EMD.

A new method, called local linear extrapolation, is introduced to suppress end effects, and the combination of adding a high frequency sinusoidal signal to, and embedding a decorrelation operator in, the process of EMD is introduced to eliminate mode mixing. This new method has good performance in restraining end effects, and it can eliminate mode mixing even if the decomposed multicomponent signal is mixed with high-frequency discontinuous signals and low-frequency ratio signals. In addition, this new method consumes less calculation time than other methods, such as EEMD.

### 3.1. The Suppression of End Effects by Local Linear Extrapolation

Based on the fundamental cause of end effects and inspired by the research achievements [28,29,38] of experts and scholars, a new method, which is called local linear extrapolation, is proposed to eliminate the problem of end effects. This method is the improved version of Yang’s method [28]. The advantage of this method is that it can determine the extremum of an endpoint according to the development trend of both ends without extending or predicting the data. The structure of the original data will not be changed with this method, so original information can be retained.

**Figure 1:**
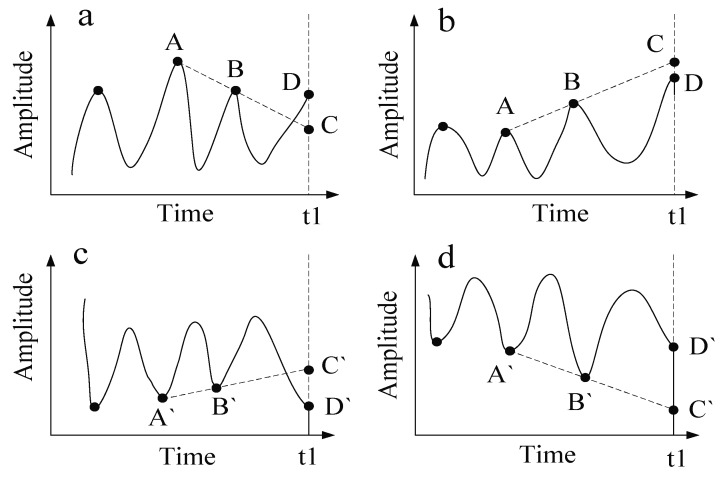
The legend for the local linear extrapolation method. (**a**) The endpoint D is greater than the intersection C; (**b**) The endpoint D is lower than the intersection C; (**c**) The endpoint D’ is lower than the intersection C’; (**d**) The endpoint D’ is greater than the intersection C’.

Taking the determination of the right extreme points as an example, the local linear extrapolation method can be expressed as follows:

(1) The determination of the right maximum point:
  (1) The local maximum value points A and B are determined;
  (2) A straight line AB is determined by point A and B. Point C is the intersection point of line AB and the time axis that corresponds to the right endpoint D, as shown in Figure 1a.
  (3) If the value of point C is less than the value of the right endpoint D, the right endpoint D is identified as the maximum value; if the value of point C is greater than the value of the right endpoint D, as shown in Figure 1b, the maximum value point is identified in the following situations: if the value of point C is greater than twice the average value of point B + A, the maximum value point is identified as half of the value of adding C to D. Otherwise, the maximum value point is identified as the intersection point C.
(2) The determination of the right minimum point:
  (1) The local maximum value points A` and B` are determined;
  (2) A straight line A`B` is determined by point A` and B`. Point C` is the intersection point of line A`B` and the time axis that corresponds to the right endpoint D`, as shown in Figure 1c.
  (3) If the value of point C` is greater than the value of the right endpoint D`, the right endpoint D` is identified as the minimum value; if the value of point C` is less than the value of the right endpoint D`, as shown in Figure 1d, the minimum value point is identified in the following situations: if the value of point C` is less than twice the average value of point B` + A`, the minimum value point is identified as half of the value of adding C` to D`. Otherwise, the minimum value point is identified as the intersection point C`.

The abovementioned points D and D` are actually the same point. In order to describe the method conveniently, we use two kinds of symbols, D and D`, when the right endpoint appears in different figures. The method for determining the left extreme points is similar to the method for determining the right extreme points.

**Figure 2:**
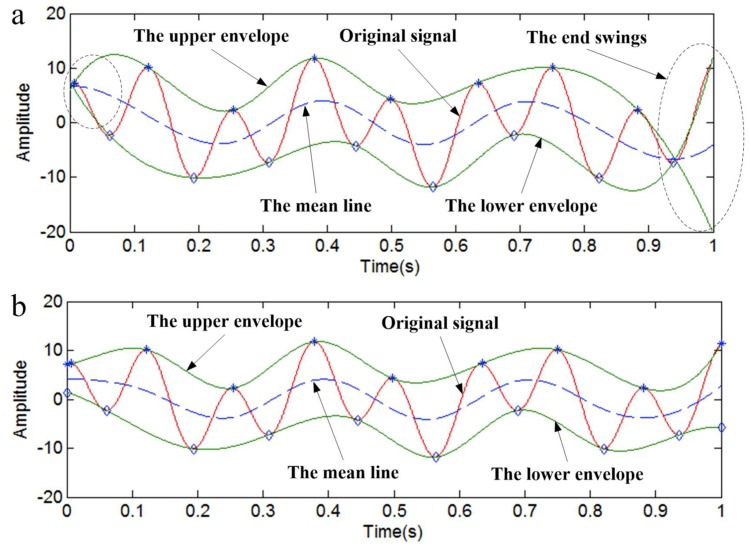
The envelopes. (**a**) The original envelopes; (**b**) The envelopes that were obtained by the local linear extrapolation method.

The envelope curve will be improved by the method of local linear extrapolation. Figure 2b shows the envelopes that are obtained by the local linear extrapolation method. Compared with Figure 2a, we can notice that the end of each envelope in Figure 2b is more stable, and the upper and lower envelopes completely include the original signal.

In order to verify the effectiveness of the local linear extrapolation method for restraining end effects, this new method is applied to analyzing the simulation signal *y*_1_(*t*). The expression of *y*_1_(*t*) is as shown in Equation (8), and the sampling frequency is set as 1024 Hz.

(8) $$y_{1}(t)=5\sin(2\pi 15t)+6\sin(2\pi 80t),t\in [0,1]$$

**Figure 3:**
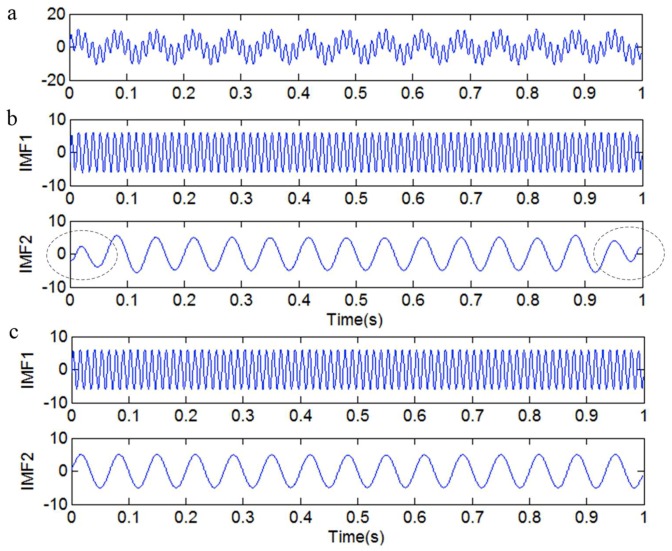
The simulated signal *y*_1_(*t*) and intrinsic mode functions (IMFs). (**a**) The simulated signal *y*_1_(*t*); (**b**) the IMFs obtained by the original empirical mode decomposition (EMD) method; (**c**) the IMFs obtained by the local linear extrapolation method.

The time-domain plot and IMFs are shown in Figure 3. The IMFs in Figure 3b are obtained by the original EMD method, and the IMFs in Figure 3c are obtained by the EMD method that has been revised by the local linear extrapolation method. It is obvious that both ends of IMF2 contain end swing in Figure 3b, but the IMFs in Figure 3c are normal.

**Figure 4:**
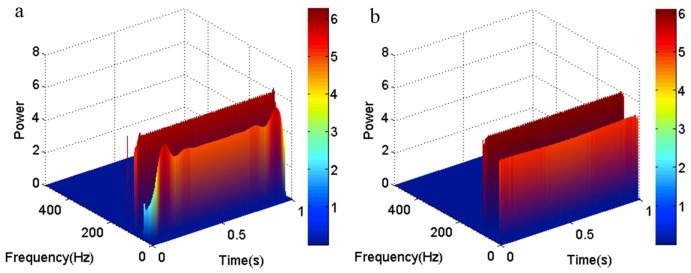
The three-dimensional time-frequency-power spectrum. (**a**) The three-dimensional time-frequency-power spectrum obtained by the original EMD method; (**b**) The three-dimensional time-frequency-power spectrum obtained by the local linear extrapolation method.

To contrast the effect of the two methods more obviously, the three-dimensional time-frequency-power spectrum of each method is shown in Figure 4. It is obvious that the time-frequency-power spectrum is mutational at the both ends of the low-frequency area, as shown in Figure 4a. The time-frequency-power spectrum in Figure 4b is stable. So, it shows that the EMD method that has been revised by the local linear extrapolation method works better than the original EMD method in eliminating end effects.

In order to verify the effectiveness of the local linear extrapolation method for decomposing a signal that contains noise, a simulation verification is also conducted. The simulated signal *y*_2_(*t*) is as shown in Equation (9), and the sampling frequency is set as 1024 Hz.

(9) $$y_{2}(t)=5\sin(2\pi 15t)+6\sin(2\pi 80t)+noise,t\in [0,1]$$

where the noise is white noise.

**Figure 5:**
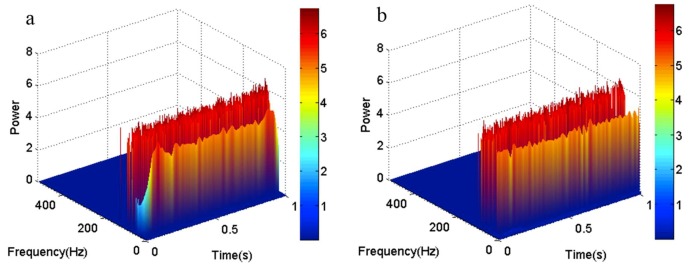
The three-dimensional time-frequency-power spectrum. (**a**) The three-dimensional time-frequency-power spectrum obtained by the original EMD method; (**b**) The three-dimensional time-frequency-power spectrum obtained by the local linear extrapolation method.

The three-dimensional time-frequency-power spectrums of each method are shown in Figure 5. Figure 5a shows that there exist end effects at the low-frequency band, but the three-dimensional plots in Figure 5b show that there are no end effects at any frequency band. This indicates that the local linear extrapolation method is better than the original EMD method for decomposing signals that contain noise.

**Figure 6:**
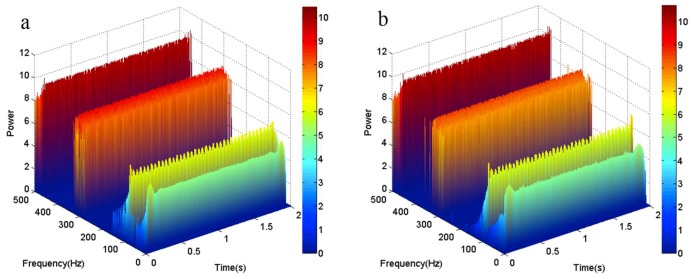
The three-dimensional time-frequency-power spectrum of the signal *y*_3_(*t*). (**a**) The three-dimensional time-frequency-power spectrum obtained by the original EMD method; (**b**) The three-dimensional time-frequency-power spectrum obtained by the local linear extrapolation method.

To verify whether the proposed method is robust with respect to different frequency bands, a synthetic signal *y*_3_(*t*), which contains multiple frequencies, is designed as shown in Equation (10). The sampling frequency is set as 4096 Hz. The three-dimensional time-frequency-power spectrums are shown in Figure 6. It can be seen from Figure 6 that, even if the signal contains different frequency bands, a relatively reliable result can be obtained by using the proposed method.

(10) $$y_{3}(t)=5\sin(2\pi 15t)+6\sin(2\pi 80t)+10\sin(2\pi 300t)+8\sin(2\pi 500t),t\in [0,2]$$

**Figure 7:**
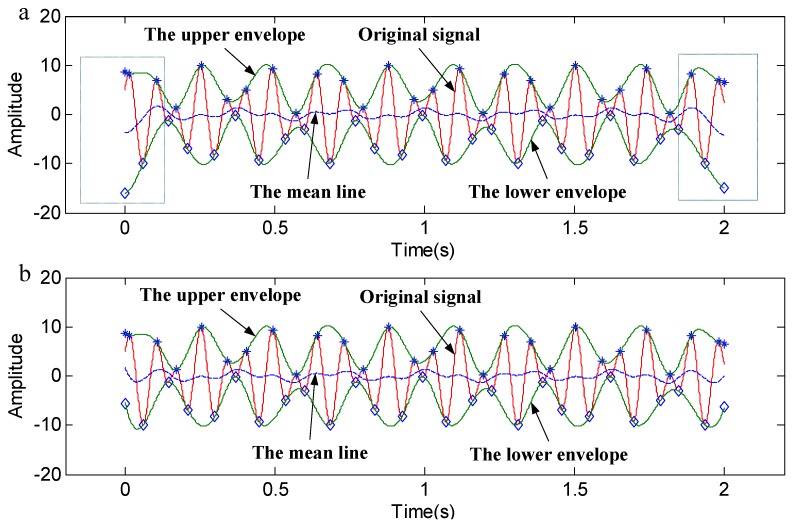
The envelopes. (**a**) The envelopes obtained by Yang’s method; (**b**) The envelopes obtained by the local linear extrapolation method.

In addition, our method is compared with Yang’s method [28]. A signal *y*_4_(*t*) is applied to analyze the advantages of our method. The envelopes that were obtained by the two methods are shown in Figure 7. Figure 7a shows the envelopes that were obtained by Yang’s method. Figure 7b shows the envelopes that were obtained by the local linear extrapolation method (the proposed method). The sampling frequency is set as 1024 Hz.

(11) $$y_{4}(t)=5\cos(2\pi 8t)+5\sin(2\pi 12.8t),t\in [0,2]$$

It can be seen from Figure 7a that, although the original signal is enveloped by the upper and lower envelopes, the trend at both ends of the signal does not match the overall trend. It can be seen from Figure 7b that the original signal is not only enveloped by the upper and lower envelopes, but also that the trend at both ends of the signal is consistent with the overall trend of the signal. This means that the proposed method is more reliable in preserving the original information of the signal.

A quantitative comparative analysis of the two methods also is conducted. Since the mean line of the upper and lower envelopes can reflect the trend of the envelopes, the mean values of the envelopes are taken as the research object. Firstly, the mean values of the envelopes for the signal *y*_4_(*t*) by taking a 2 s window and a 3 s window are calculated, respectively. Then, the root mean square error (RMSE) of the mean values for the 2 s window in comparison to the 3 s one is calculated. The above steps were repeated for Yang’s method presented in [28]. The RMSE value of each method is shown in Table 1.

It is clear from Table 1 that the mean line of the envelopes that was obtained by the proposed method shows a smaller degree of dispersion, which means that the proposed method is more reliable. The reliability is increased by 53.3%.

### 3.2. The Suppression of Mode Mixing by Adding a High-Frequency Sinusoidal Signal and Embedding a Decorrelation Operator

Mode mixing causes aliasing in the time-frequency distribution, and it also makes individual IMFs lack physical meaning and physical uniqueness, which has a bad influence on the results of the data analysis. In order to eliminate mode mixing, a new method is proposed in this article. This method is a combination of adding a high-frequency signal and embedding a decorrelation operator.

**Figure 8:**
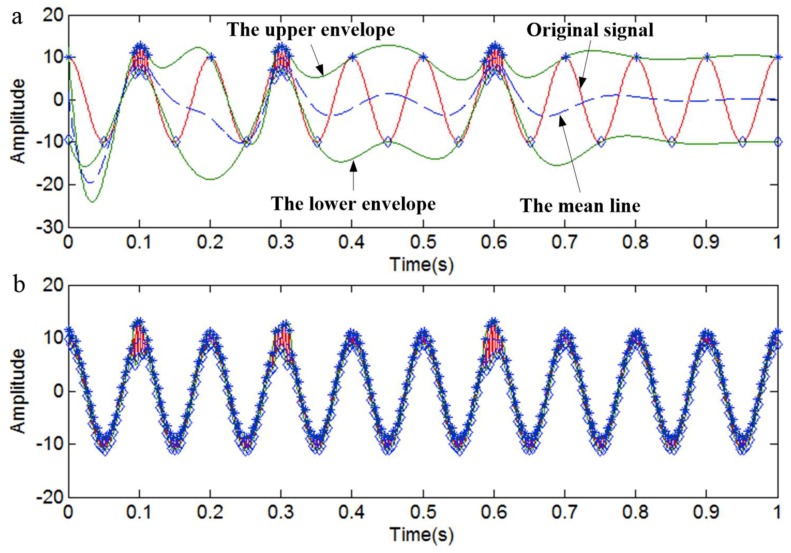
The envelopes. (**a**) The envelopes of a simulated signal; (**b**) The envelopes after adding a high-frequency sinusoidal signal.

The envelope will be changed if a signal is mixed with a discontinuous signal. It is easy to cause overshoots and undershoots, since the envelopes are mutational at the junction of two signals, as shown in Figure 8a. On the other hand, the envelopes are shared by the continuous and discontinuous signals, so the problem of mode mixing is inevitable in the EMD process. The extreme points of the signal will be redistributed, and the ‘mutational event’, which is caused by singular signal, will be inconspicuous if we add a high-frequency sinusoidal signal to the original signal. Although the envelope will also be changed, its trend will become more in accordance with the original signal, as shown in Figure 8b. So, we can obtain better decomposition effects.

As discussed by Huang et al. [6] and Peng et al. [39], the first IMF always contains a wide range of frequencies rather than a single component signal. Since the arrangement of IMFs is from high frequency to low frequency, the frequency of the added sinusoidal signal should be larger than the highest frequency of the original signal so that we can ensure that the sinusoidal signal is obtained as the first IMF. In actual fault diagnosis, the frequency of the added sinusoidal signal should be determined based on the upper limit of the analyzed frequency. The sinusoidal signal and the discontinuous signal are mixed together in the first IMF, so we can obtain the discontinuous signal by subtracting the sinusoidal signal from IMF1.

The critical problem of the sinusoidal-signal-aided method is how to determine the amplitude and frequency of the added signal. The upper limit frequency is usually used as the sinusoidal signal’s frequency. If the amplitude of the added sine signal is too small, it will not optimize the distribution of each extreme point, so mode mixing will not be eliminated. However, if the added signal’s amplitude is too large, the power of the original signal will be changed significantly, and it also will be harmful to the results of the analysis.

Xu et al. [40] introduced the normalized mean square error (NMSE) to judge the deviation of each ${imf}_{i}(t)$ and the single-frequency signal $f_{i}(t)$. For a given signal.

$x(t)=\sum_{i=1}^{N}f_{i}(t)$, the expression of NMSE is shown in Equations (12) and (13).

(12) $$\mathrm{NMSE}=\max({\mathrm{NMSE}}_{i},i=1,2,3...,N)$$

(13) $${\mathrm{NMSE}}_{i}=\frac{\int_{0}^{T}{\left|f_{i}(t)-{imf}_{i}(t)\right|}^{2}dt}{\int_{0}^{T}{\left|f_{i}(t)\right|}^{2}dt}$$

where $f_{i}(t)$ is the single-frequency component of the mixed signal *x*(*t*), and ${imf}_{i}(t)$ is the intrinsic mode functions that corresponding to $f_{i}(t)$.

**Figure 9:**
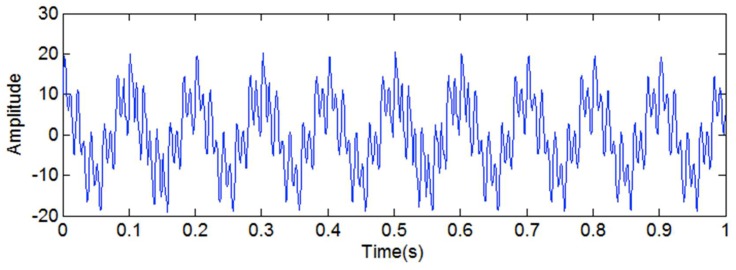
The simulated signal *y*_3_(*t*).

In this article, when it comes to the discontinuous signal, we mainly discuss the situation in which the frequency of the discontinuous signal is the highest in all of the frequency components. In order to determine the best scope of the amplitude and frequency of the added high-frequency sinusoidal signal, a simulated signal *y*_5_(*t*) is introduced. Figure 9 shows the simulated signal *y*_5_(*t*). The sampling frequency is set as 1024 Hz.

(14) $$\begin{array}{ll} y_{5}(t) & =5\sin(2\pi 50t)+6\sin(2\pi 100t)\\ & +10\cos(2\pi 10t)+y_{6}(t),t\in \left[0,1\right] \end{array}$$

(15) $$\begin{array}{ll} y_{6}(t)= & 3\sin(2\pi 200t)[u(t-0.08)-u(t-0.12)]+\\ & 3\sin(2\pi 200t)[u(t-0.28)-u(t-0.32)]+\\ & 3\sin(2\pi 200t)[u(t-0.58)-u(t-0.62)] \end{array}$$

where *u*(*t*) denotes the unit step function. *y*_6_(*t*) is a discontinuous signal.

Sinusoidal signals with different frequencies, which range from 180 Hz to 350 Hz, and different amplitudes, which range from 10 to 220, are added to the signal *y*_3_(*t*), respectively. Then, the mixed signal is decomposed by EMD. We can obtain the distribution of the NMSE according to Equations (12) and (13).

**Figure 10:**
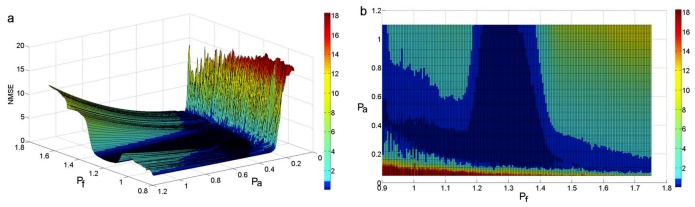
The distribution of the NMSE. (**a**) The three-dimensional distribution of the NMSE; (**b**) The distribution plan of the NMSE.

The distribution of the NMSE corresponding to different frequencies and amplitudes is shown in Figure 10. The color bar on the right denotes the NMSE value. In order to find the relationship between the added signal and the original signal, in the following figures the horizontal axis (P_f_) denotes the proportion of the added signal’s frequency and the highest frequency of the original signal. The vertical axis (P_a_) denotes the proportion of the added signal’s amplitude and the original signal’s amplitude.

**Figure 11:**
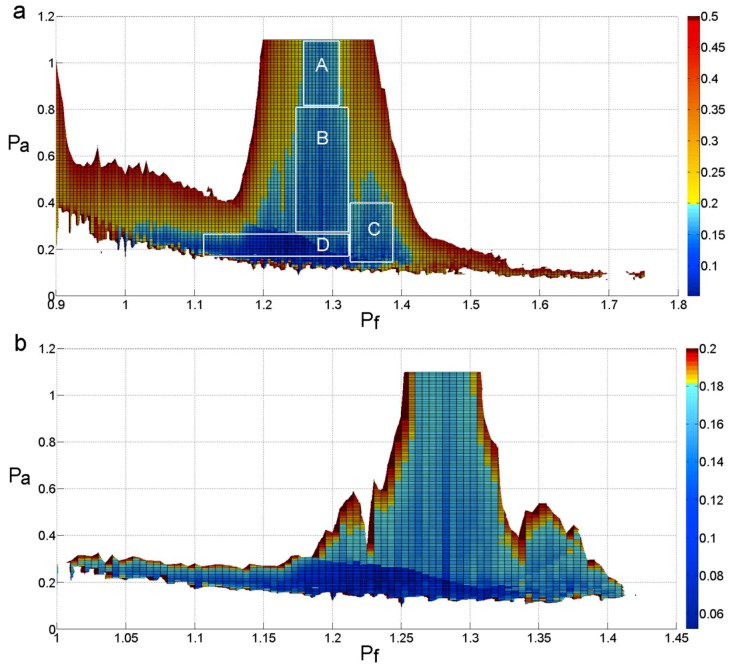
The distribution of the NMSE. (**a**) The distribution of the NMSE that less than 0.5; (**b**) The distribution of the NMSE that less than 0.2.

For the purposes of selecting a reasonable range of frequency and amplitude, we will narrow the scope of the NMSE. The distribution of the NMSE that is less than 0.5 is shown in Figure 11a. The color bar in the figures denotes the value of each NMSE. Figure 11a shows that the NMSE value in areas A, B, and C is less than 0.2, and the value in area D is less than 0.1. Theoretically, any (P_f_, P_a_) pair in areas A, B, C, and D can be selected. Figure 11b shows all of the areas in which the NMSE value is less than 0.2. A detailed analysis will be carried out for the purpose of choosing a more precise range of frequency and amplitude.

**Figure 12:**
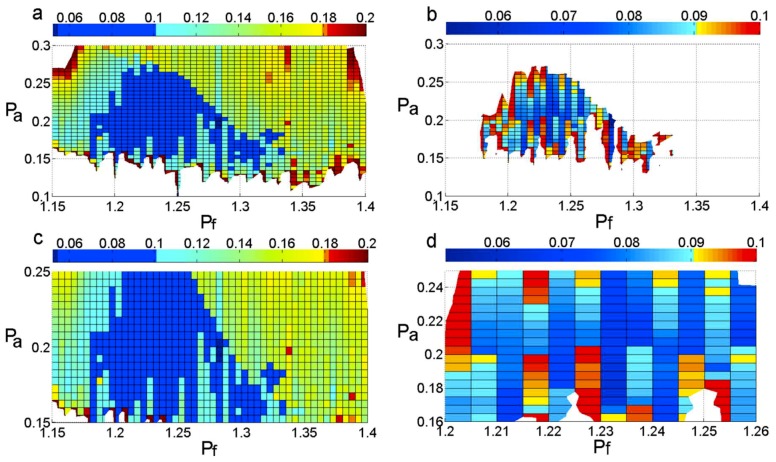
The distribution of the NMSE. (**a**,**c**) show the NMSE values that are less than 0.2; (**b**,**d**) show the NMSE values that are less than 0.1.

As shown in Figure 12a, when the frequency of the added signal is determined to be 1.15~1.4 times the original signal’s highest frequency and the amplitude of the added signal is determined to be 0.1~0.3 times the original signal’s peak, not all of the NMSE values are less than 0.2, and, in this range, the NMSE values that are less than 0.1 occupy a small proportion, as shown in Figure 12b.

Figure 12c illustrates that, when the frequency is determined to be 1.15~1.4 times the original signal’s highest frequency and the amplitude is determined to be 0.15~0.25 times the original signal’s peak, almost all of the NMSE values are less than 0.2, and, in this area, the NMSE values that are less than 0.1 occupy almost half of the proportion. When the frequency is determined to be 1.2~1.26 times the original signal’s highest frequency and the amplitude is determined to be 0.16~0.25 times the original signal’s peak, almost all of the NMSE values are less than 0.1, as shown in Figure 12d.

In this paper, in order to expand the range of choices of frequency and amplitude, the frequency of the added signal is determined to be 1.15~1.4 times the original signal’s highest frequency, and the amplitude is determined to be 0.15~0.25 times the original signal’s peak.

For the purpose of obtaining the amplitude and frequency information of the original signal, we apply a Fourier transform to determine the highest frequency of the signal. Although there are some limitations in terms of nonlinear signal processing by a Fourier transform, we just found the approximate frequency here. In order to determine the peak value of the original signal, first we find all of the local maximum values, and then take the average value of the local maximums as the peak value.

**Figure 13:**
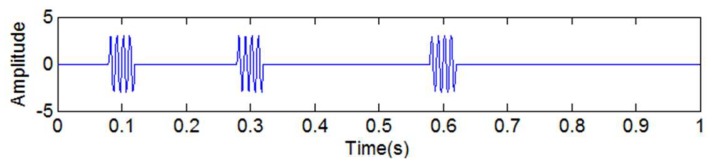
The simulated discontinuous high-frequency signal *y*_8_(*t*).

In order to verify the effectiveness of the proposed method, we take the mixed signal *y*_7_(*t*) as an example. The sampling frequency is set as 1024 Hz.

(16) $$y_{7}(t)=10\cos(2\pi 10t)+y_{8}(t),t\in [0,1]$$

(17) $$\begin{array}{ll} y_{8}(t)= & 3\sin(2\pi 200t)[u(t-0.08)-u(t-0.12)]+\\ & 3\sin(2\pi 200t)[u(t-0.28)-u(t-0.32)]+\\ & 3\sin(2\pi 200t)[u(t-0.58)-u(t-0.62)] \end{array}$$

where *u*(*t*) denotes the unit step function, and *y*_8_(*t*) is a discontinuous high-frequency signal, as shown in Figure 13.

The added sinusoidal signal *y*_9_(*t*) is determined as

(18) $$y_{9}(t)=2\sin(2\pi 230t),t\in [0,1]$$

**Figure 14:**
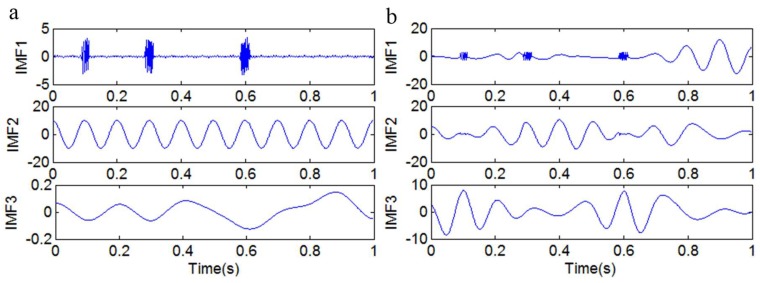
The first three IMFs. (**a**) The IMFs obtained by adding the sinusoidal signal *y*_9_(*t*); (**b**) the IMFs obtained by the original EMD method.

Figure 14 shows the IMFs that were obtained by different EMD methods. Figure 14a illustrates that mode mixing is eliminated by adding the sinusoidal signal *y*_7_(*t*). Although there are illusive components, we can choose the main IMFs by the method presented in Section 3.3. However, there exists mode mixing in Figure 14b, in which the IMFs have been obtained by the original EMD method.

**Figure 15:**
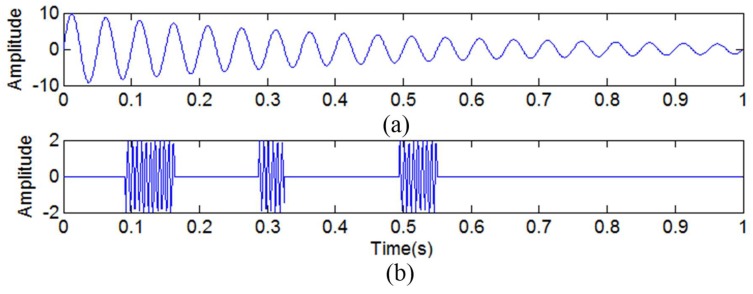
The simulated signals. (**a**) The simulated signal *y*_11_(*t*); (**b**) the simulated signal *y*_12_(*t*).

Another simulated nonlinear signal *y*_10_(*t*) can also be used to verify the effectiveness of the proposed method. The sampling frequency is set as 1024 Hz.

(19) $$y_{10}(t)=y_{11}(t)+y_{12}(t),t\in \left[0,1\right]$$

where $y_{11}(t)=10\cdot e^{-2t}\cdot \sin(40\pi t)$ is a nonlinear decay signal, as shown in Figure 15a.

(20) $$\begin{array}{ll} y_{12}(t)= & 2\sin(2\pi 150t)[u(t-0.09)-u(t-0.16)]+\\ & 2\sin(2\pi 150t)[u(t-0.28)-u(t-0.33)]+\\ & 2\sin(2\pi 150t)[u(t-0.49)-u(t-0.55)] \end{array}$$

where *u*(*t*) denotes the unit step function, and *y*_12_(*t*) is a discontinuous high-frequency signal, as shown in Figure 15b.

The added sinusoidal signal *y*_13_(*t*) is determined as

(21) $$y_{13}(t)=1.25\sin(2\pi 180t),t\in [0,1].$$

**Figure 16:**
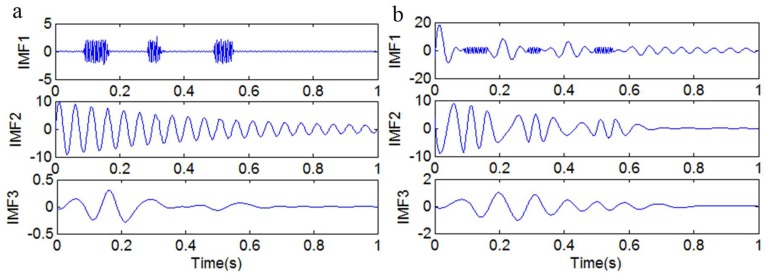
The first three IMFs. (**a**) The IMFs obtained by adding the sinusoidal signal *y*_13_(*t*); (**b**) the IMFs obtained by the original EMD method.

Figure 16a shows the IMFs that were obtained by adding the sinusoidal signal *y*_13_(*t*). It is obvious that the mixed signal *y*_10_(*t*) can be decomposed into a discontinuous high-frequency signal *y*_12_(*t*) and a nonlinear decay signal *y*_11_(*t*). The IMFs in Figure 16b, which have been obtained by the original EMD method, contain serious mode mixing.

Although adding a high-frequency sinusoidal signal to the original signal can eliminate mode mixing when the signal is mixed with a high-frequency discontinuous signal, it is still useless to signals that are mixed with low-frequency ratio signals. The fundamental cause of mode mixing lies in the process of decomposition being not strictly orthogonal in EMD. In order to eliminate mode mixing, we should make sure that the IMFs are orthogonal to each other. Taking two random variables as an example, the covariance is as shown in Equation (22):

(22) $$C_{xy}=E\left\{\left(x-E(x))(y-E(y)\right)\right\}=E(xy)-E(x)E(y)$$

where

x=x(n),y=y(n), and *n* denotes the data length.

According to Equation (22), we can obtain the following conclusions:

(1) If $C_{xy}=0$, then x(n) and y(n) are uncorrelated;
(2) If E(xy)=0, then x(n) and y(n) are orthogonal;
(3) Obviously, if x(n) and y(n) are uncorrelated ($C_{xy}=0$) and E(x)=0, E(y)=0, then x(n) and y(n) are orthogonal.

From the above analysis, we obtain the conclusion that the orthogonality equals to x(n) and y(n) being uncorrelated for zero mean random variables. According to Section 2, we know that the IMF satisfies the zero mean features. So the decorrelation operator [36] is introduced during the EMD process. A correlation coefficient ‘*r*’ is defined as in Equation (23)

(23) $$r=\frac{y^{T}(n)\cdot x(n)}{y^{T}(n)\cdot y(n)}.$$

Obviously, r⋅y(n) represents the cross-correlation part of x(n) and y(n). The data v(n), which is not correlated with y(n), can be obtained by Equation (24).

(24) v(n)=x(n)−r⋅y(n)

According to the definition of the correlation coefficient, r should satisfy the condition |r|≤1. Sometimes, it is difficult to satisfy this condition due to the difference in each IMF’s amplitude. If we normalize x(n) and y(n), and assume that $r_{N}$ is the correlation coefficient under a normalized condition, we can draw the conclusion that the correlation coefficient r is just the scaling of $r_{N}$, and it does not influence the result of removing the relevant parts [36]. According to the abovementioned analysis, a new method is proposed to eliminate mode mixing, which is expressed as follows:

(1) Find all of the local maximum values of the original signal, and then take the average value of the local maximums as the peak value. Apply the fast Fourier transform to determine the highest frequency of the original signal. According to the highest frequency and the peak value of the original signal, the high-frequency sinusoidal signal $\hat{\overset{⌢}{x}}(t)$ is determined (the frequency of the added signal is determined to be 1.15~1.4 times the original signal’s highest frequency and the amplitude is determined to be 0.15~0.25 times the original signal’s peak).

(2) Adding the high-frequency sinusoidal signal $\hat{\overset{⌢}{x}}(t)$ to the original signal *X*(*t*), we can obtain a mixed-signal X′(t). Then, the new signal X′(t) is processed by EMD. Finally, a series of IMFs $c_{i}(t)$ can be obtained.

(3) The first IMF ${\bar{c}}_{1}(t)$ without the added high-frequency sinusoidal signal $\hat{\overset{⌢}{x}}(t)$ can be obtained by the following equation

(25) $${\bar{c}}_{1}(t)=c_{1}(t)-\hat{\overset{⌢}{x}}(t).$$

(4) Calculate the correlation coefficient $r_{1}$ between ${\bar{c}}_{1}(t)$ and $c_{2}(t)$. Then, we can obtain the IMF ${\hat{c}}_{1}(t)$ by the following equation

(26) $${\hat{c}}_{1}(t)={\bar{c}}_{1}(t)-r_{1}\cdot c_{2}(t).$$

(5) Reserve ${\hat{c}}_{1}(t)$. Then, we can obtain $\hat{X}(t)$ by the equation $\hat{X}(t)=X\prime (t)-{\hat{c}}_{1}(t)-\hat{\overset{⌢}{x}}(t)$. Decomposing $\hat{X}(t)$ by EMD, we get a series of IMFs ${\tilde{c}}_{i}(t)$. Calculate the correlation coefficient ${\bar{r}}_{1}$ between ${\hat{c}}_{1}(t)$ and ${\tilde{c}}_{1}(t)$. If ${\bar{r}}_{1}$ is less than the threshold δ, ${\hat{c}}_{1}(t)$ is defined as the first IMF; that is, IMF1. Otherwise, repeat the decorrelation operation between ${\hat{c}}_{1}(t)$ and ${\tilde{c}}_{i}(t)$.

(6) Repeat the decorrelation operation until all of the correlation coefficients between the IMFs are less than the threshold δ. Finally, we can obtain a series mutually orthogonal IMFs.

**Figure 17:**
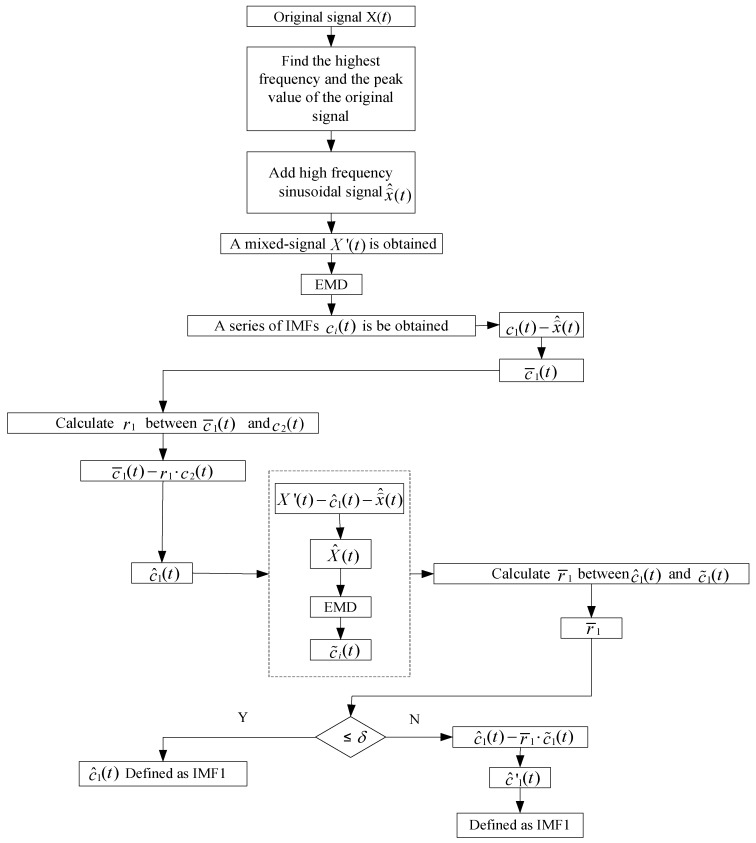
The flow chart for eliminating mode mixing.

It is worth noting that, because the discontinuous high-frequency signal has been separated as IMF1 from the mixed signal, the high-frequency sinusoidal signal is unnecessary when decomposing the remaining IMFs. The block diagram of the first five steps is shown in Figure 17.

**Figure 18:**
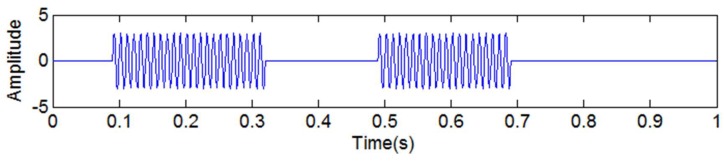
The simulated discontinuous signal *y*_15_(*t*).

In order to validate the effectiveness of the method, the mixed signal *y_1_*_4_(*t*), which contains both a high-frequency interval signal and low-frequency ratio signals, is taken as an example to be decomposed by the above method. The sampling frequency is set as 1024 Hz.

(27) $$y_{14}(t)=10\cos(2\pi 10t)+5\sin(2\pi 50t)+6\sin(2\pi 80t)+y_{15}(t),t\in [0,1]$$

(28) $$\begin{array}{ll} y_{15}(t)= & 3\sin(2\pi 200t)[u(t-0.09)-u(t-0.32)]+\\ & 3\sin(2\pi 200t)[u(t-0.49)-u(t-0.69)] \end{array}$$

where *u*(*t*) denotes the unit step function, and *y*_15_(*t*) is a discontinuous signal, as shown in Figure 18. The added signal *y*_16_(*t*) is as shown in Equation (29).

(29) $$y_{16}(t)=3\sin(2\pi 232t),t\in [0,1]$$

**Figure 19:**
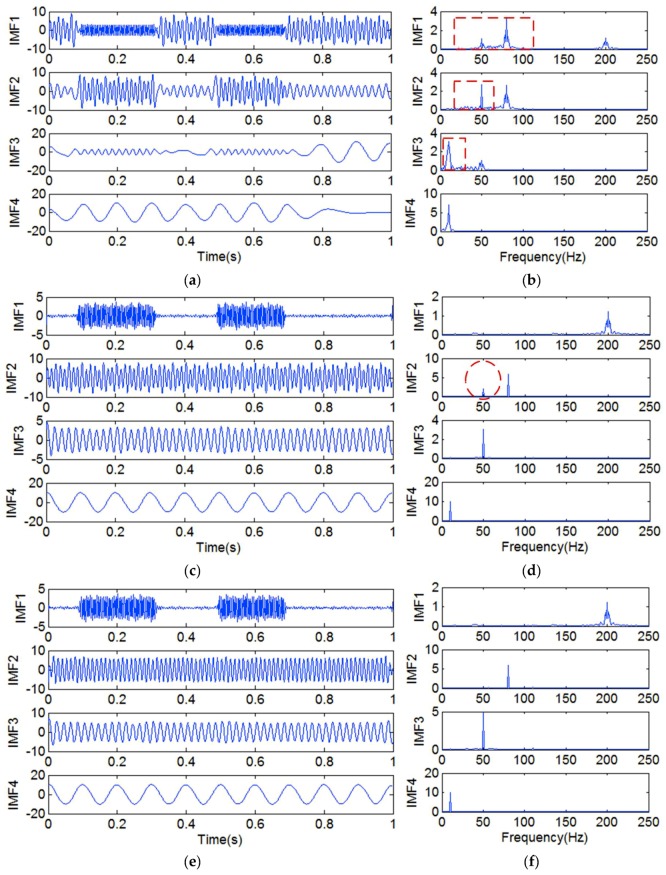
The IMFs and frequency spectra. (**a**) The IMFs obtained by the original EMD method; (**b**) the frequency spectrum corresponding to each IMF in (**a**); (**c**) the IMFs obtained by only adding a high-frequency sinusoidal signal *y*_1__6_(*t*); (**d**) the frequency spectrum corresponding to each IMF in (**c**); (**e**) the IMFs obtained by adding a high-frequency sinusoidal signal *y*_1__6_(*t*) and embedding a decorrelation operator; (**f**) the frequency spectrum corresponding to each IMF in (**e**).

The results of each decomposition method are shown in Figure 19. Figure 19a shows the IMFs that were obtained by the original EMD method, and Figure 19b shows the frequency spectrum corresponding to each IMF in Figure 19a. It is obvious that there is serious mode mixing in each IMF that was obtained by the original EMD method. Figure 19c shows the IMFs that were obtained only by adding a high-frequency sinusoidal signal, and it illustrates that the discontinuous signal can be extracted, but that there is mode mixing in IMF2. As shown in Figure 19d, IMF2 not only contains the frequency component of 80 Hz, but also contains the frequency component of 50 Hz. Figure 19e shows the IMFs that were obtained by adding a high-frequency sinusoidal signal and embedding the decorrelation operator, and it is obvious that all four kinds of signals are extracted without mode mixing. So, the method of adding a high-frequency sinusoidal signal to, and embedding a decorrelation operator in, EMD is effective in decomposing a signal that is mixed with a high-frequency discontinuous signal and low-frequency ratio signals.

**Figure 20:**
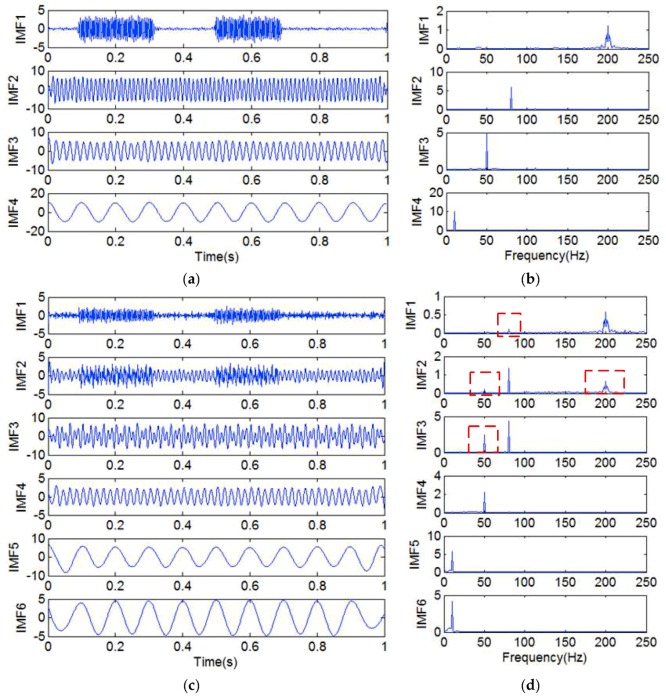
(**a**) The IMFs obtained by adding the high-frequency sinusoidal signal *y*_14_(*t*) and embedding a decorrelation operator; (**b**) the frequency spectrum corresponding to each IMF in (**a**); (**c**) the IMFs obtained by the ensemble empirical mode decomposition (EEMD) method; (**d**) the frequency spectrum corresponding to each IMF in (**c**).

This proposed method is also compared with the EEMD method. We also take the signal *y*_14_(*t*) as the experimental signal. In the EEMD method, an ensemble size of 1000 is used, and the added white noise in each ensemble member has a standard deviation of 0.2 [31]. The IMFs that were obtained by the two methods are shown in Figure 20.

Figure 20a,b show the IMFs and frequency spectra that were obtained by adding the high-frequency sinusoidal signal *y*_16_(*t*) and embedding a decorrelation operator. Figure 20c,d show the IMFs and frequency spectra that were obtained by the EEMD method. From Figure 20c,d, we can notice that mode mixing exists in IMF1, IMF2, and IMF3, as shown in the dashed box of Figure 20d. However, Figure 20a,b illustrate that the single-frequency signals are decomposed without mode mixing. This shows that the proposed method is better than EEMD for eliminating mode mixing.

Table 2 compares the computational time by a computer with a 3.20 GHz processor (Intel Core i5-4460). In order to eliminate the influence of accidental factors, we decomposed the same data five times (denoted A, B, C, D, and E), then calculated the average computation time value.

Table 2 shows that the average computation time of the EEMD method is 118.06 s. By contrast, the average computation time of the revised EMD is 0.55 s, which is far less than the EEMD method, and this advantage is more suitable for real-time monitoring and diagnosis than EEMD.

Although the computation time of EEMD will be reduced by reducing the ensemble number, the decomposition effect will be worse. In general, an ensemble number of a few hundred will lead to a very good result. This means that the time EEMD consumes is still hundreds of times that of the proposed method.

### 3.3. The Selection of IMF Components

A pseudo intrinsic mode function is inevitable during the EMD process, and it will bring bad effects to the results of the analysis. Real intrinsic mode functions correlate well with the original signal, but a pseudo-component will not [11]. So, the correlation coefficients between the IMFs and the original signal are introduced to choose the real IMFs. The steps are expressed as follows [11]:

(1) The threshold is determined. Calculate the correlation coefficient between each IMF and the original signal.
(2) The IMF will be eliminated if the correlation coefficient between the IMF and the original signal is less than the threshold; otherwise, the IMF will be reserved.
(3) Rearrange the reserved IMFs according to the frequency from high to low.

In order to analyze the influence of pseudo intrinsic mode functions on the results of the analysis and verify the effectiveness of the IMF selection method, a simulated mixed signal *y*_17_(*t*) is processed by two methods: one method reserves the pseudo intrinsic mode functions and the other method eliminates them according to the correlation coefficients. The sampling frequency is set as 1024 Hz.

(30) $$y_{17}(t)=5\sin(2\pi 50t)+6\sin(2\pi 80t),t\in [0,1]$$

Table 3 shows the correlation coefficients between each IMF and the original signal. It is obvious that the first two IMFs have a strong correlation with the original signal. The correlations of the other IMFs with the original signal are weaker.

**Figure 21:**
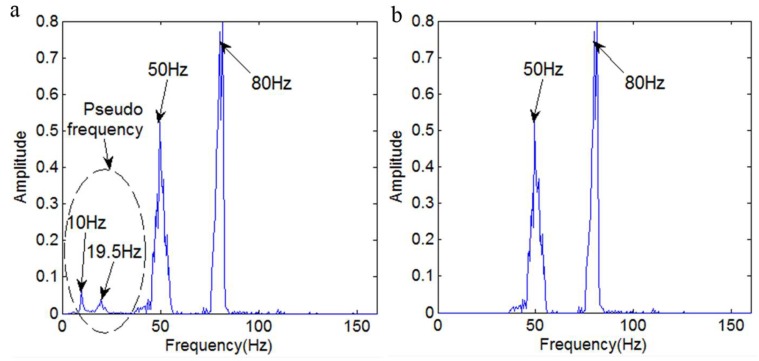
The marginal spectrum. (**a**) The marginal spectrum obtained without eliminating the pseudo frequency; (**b**) The marginal spectrum obtained by eliminating the pseudo frequency.

Figure 21 shows the marginal spectrum of the signal *y*_17_(*t*). Figure 21a shows the marginal spectrum obtained without eliminating the pseudo intrinsic mode functions. The marginal spectrum in Figure 21b is obtained by eliminating the pseudo intrinsic mode functions.

Figure 21a illustrates that there are four main frequencies: 80 Hz, 50 Hz, 19.5 Hz, and 10 Hz. Obviously, the 19.5 Hz and 10 Hz frequencies are not contained in the signal *y*_17_(*t*), so they are determined to be pseudo-components. Figure 21b illustrates that it only contains the two frequencies 80 Hz and 50 Hz, which is consistent with the frequency components of the signal *y*_17_(*t*). It is clear that the IMF selection method is effective.

## 4. Fault Diagnosis in a Rotor System by the Revised HHT Method

**Figure 22:**
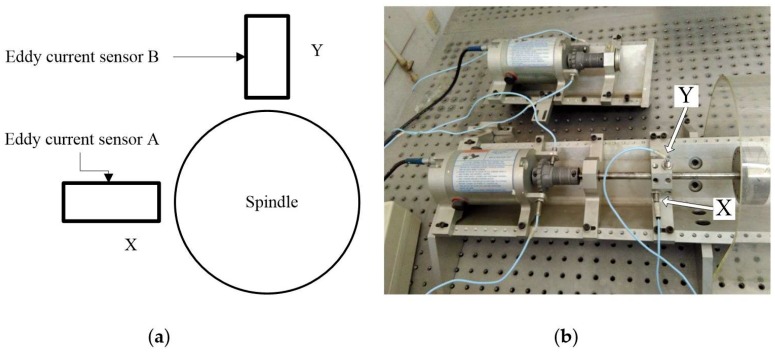
The experimental facilities. (**a**) The arrangement of the eddy current sensors; (**b**) The experimental setup.

In order to verify the effectiveness of this revised HHT method with respect to actual fault diagnosis, an experiment was carried out on a rotor test bench. The vibration displacement signals under normal, rubbing, and misalignment conditions were collected by two mutually vertical eddy current sensors. Figure 22 shows a sketch of the eddy current sensors and the experimental field. The sampling frequency is set at 1024 Hz, and the rotation speed is set at 960 rpm, so we can know that the fundamental frequency of the rotation speed is 16 Hz. The signals are denoised during the process of collection so that we can ignore the influence of stochastic noise.

**Figure 23:**
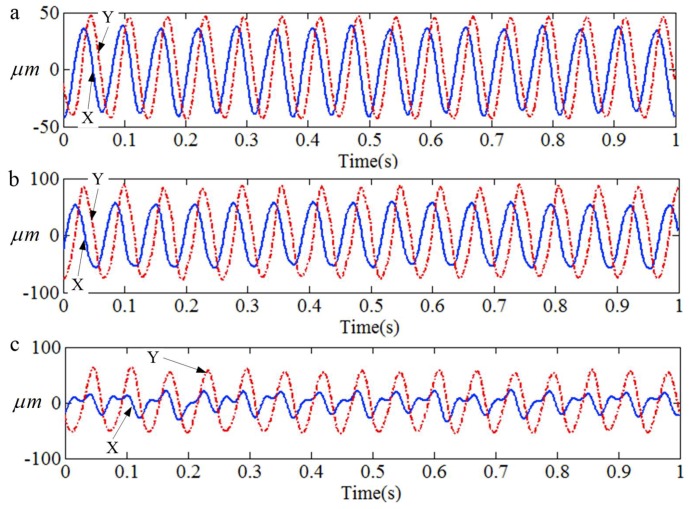
The vibration displacement signals under different running conditions. (**a**) Normal; (**b**) Rubbing; (**c**) Misalignment.

The vibration displacement signals of the X direction and the Y direction under different conditions are shown in Figure 23. Figure 23a–c illustrate the vibration displacement signals under the normal, rubbing, and misalignment conditions, respectively. It is difficult to find the difference between the normal and rubbing conditions according to the original displacement signals.

**Figure 24:**
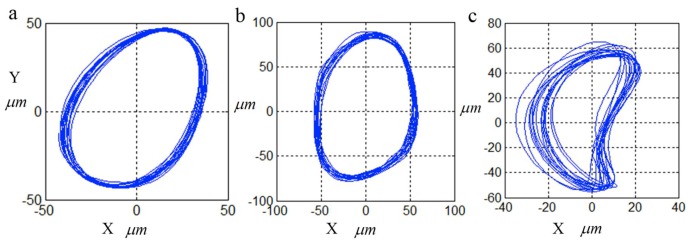
The axis orbits under different running conditions. (**a**) Normal; (**b**) Rubbing; (**c**) Misalignment.

By combining the vibration displacement signals of the X direction and the Y direction, the axis orbit of each running status can be obtained. The axis orbits under the normal, rubbing and misalignment conditions are shown in Figure 24. Figure 24 illustrates that the axis orbits of the misalignment are different from the normal running status and the rubbing running status, but it still fails to distinguish between the normal status and the rubbing status.

The original HHT and revised HHT are applied to analyze the vibration displacement signals of the normal, rubbing, and misalignment running states, respectively. The displacement signal of the X direction is selected as the signal to be analyzed. In the revised HHT, the added auxiliary signal *y*_18_(*t*) is determined as follows:

(31) $$y_{18}(t)=2.5\sin(2\pi 50t),t\in [0,1]$$

**Figure 25:**
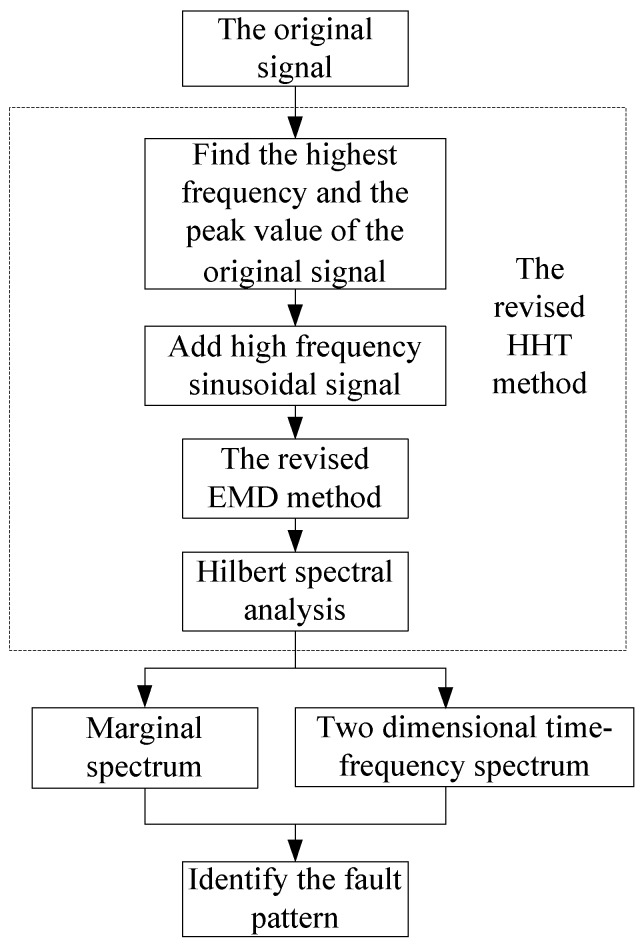
The flow chart for signal analysis. HHT, Hilbert–Huang transform.

Figure 25 shows the flow chart of signal processing.

**Figure 26:**
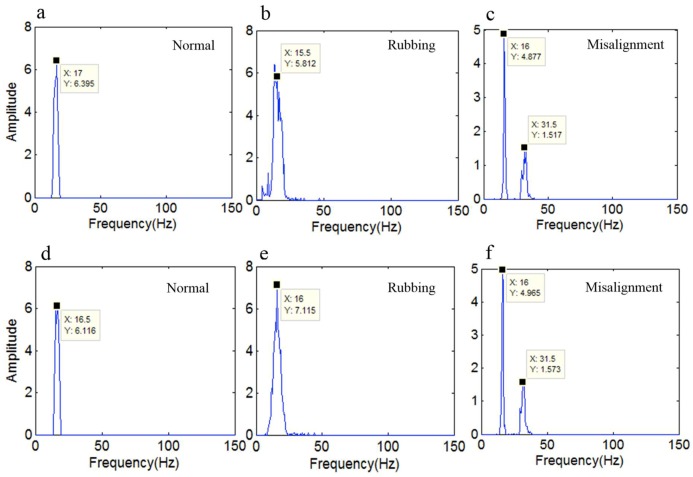
The marginal spectrum under different running states. (**a**–**c**) show the marginal spectrum of the normal, rubbing, and misalignment running states obtained by the original HHT; (**d**–**f**) show the marginal spectrum of the normal, rubbing, and misalignment running states obtained by the revised HHT.

Figure 26 shows the marginal spectrum of different running states that was obtained by the original HHT and the revised HHT method, respectively. Figure 26a–c shows the marginal spectrum of the normal, rubbing, and misalignment running states that was obtained by the original HHT; Figure 26d–f shows the marginal spectrum of the normal, rubbing, and misalignment running states that was obtained by the revised HHT. Figure 26a shows that the frequency of the normal running state is 17 Hz, while Figure 26d shows that the frequency of the normal running state is 16.5 Hz, which is closer to the theoretic frequency. Figure 26b,e shows that the frequency of the rubbing running state is 15.5 Hz and 16 Hz, respectively, which is similar to the frequency of the normal running state. Figure 26c,f shows there are two kinds of frequency (16 Hz and 31.5 Hz) when the rotor system is in misalignment. Obviously, we can recognize misalignment by the marginal spectrum, but it still fails to recognize the normal and rubbing running states only by the marginal spectrum. So, the time-frequency spectrum is applied to distinguish the normal, rubbing, and misalignment running states.

**Figure 27:**
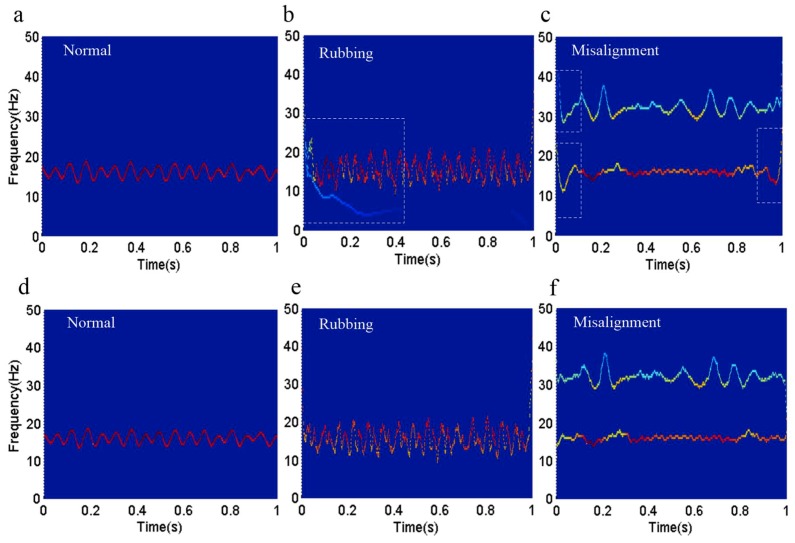
The time-frequency spectrum under different running states. (**a**–**c**) show the time-frequency spectrum of the normal, rubbing, and misalignment running states obtained by the original HHT; (**d**–**f**) show the time-frequency spectrum of the normal, rubbing, and misalignment running states obtained by the revised HHT.

The time-frequency spectrum can reflect the regularity of change in the frequency when the rotor system is running. Figure 27 shows the time-frequency spectrum of different running states that were obtained by the original HHT and the revised HHT, respectively.

Figure 27a,d shows the time-frequency spectrum of the normal running state. The time-frequency spectrum in Figure 27a was obtained by the original HHT, and the time-frequency spectrum in Figure 27d was obtained by the revised HHT. Figure 27a,d illustrate that the instantaneous frequency of the normal running state floats around 16 Hz.

Figure 27b,e show the time-frequency spectrum of the rubbing running state. The time-frequency spectrum in Figure 27b was obtained by the original HHT, and the time-frequency spectrum in Figure 27e was obtained by the revised HHT. Figure 27b,e illustrate that the instantaneous frequency of the rubbing running state floats around 16 Hz. However, the time-frequency spectrum shows a wider frequency band than the normal running state. Additionally, the waveform of the rubbing running state is irregular. It is obvious that the time-frequency spectrum in Figure 27b has the problem of end effects and mode mixing. In Figure 27e, the problem of end effects is negligible. According to the comparison in Figure 27b,e, we can see that the revised HHT is better than the original.

Figure 27c,f shows the time-frequency spectrum of the misalignment running state. The time-frequency spectrum in Figure 27c was obtained by the original HHT, and the time-frequency spectrum in Figure 27f was obtained by the revised HHT. Figure 27c,f illustrate that the instantaneous frequency of the misalignment running state floats around 16 Hz and 32 Hz. However, there are end effects in Figure 27c. Finally, based on the research and analysis above, we can draw the conclusion that, according to the time-frequency spectrum, we can identify the different running states (i.e., normal, rubbing, and misalignment). What is more, the revised HHT is more effective than the original.

Table 4 compares the computational time of each method by a computer with a 3.20 GHz processor (Intel Core i5-4460). Table 4 shows that the average computation time of the proposed method and the original method is 0.95 s and 1.29 s, respectively. It can be seen from Table 4 that the proposed method consumes more time than the original method. This is probably due to the fact that, in the proposed method, the steps of embedding the decorrelation operator and determining the frequency and amplitude of the added signal will take some time; however, the difference in time consumed between the two methods is not obvious, and it can be ignored in practice.

## 5. Conclusions

In this paper, a revised HHT is proposed, and the proposed HHT is applied to detect the running status of a rotor system. The results show that the proposed method is effective with respect to fault diagnosis. The following conclusions can be drawn from this work:

(1) In order to eliminate end effects and mode mixing, a revised HHT is proposed. The local linear extrapolation method is introduced to suppress end effects. The combination of adding a high-frequency sinusoidal signal to, and embedding a decorrelation operator in, the process of EMD is introduced to eliminate mode mixing.
(2) With respect to eliminating end effects, the local linear extrapolation method can determine the extremum of an endpoint according to the development trend of both ends without extending or predicting the data. The structure of the original data will not be changed with this method, so more original information can be retained.
(3) With respect to eliminating mode mixing, the method of combining a high-frequency sinusoidal signal and a decorrelation operator has excellent performance in decomposing a multicomponent signal that is mixed with high-frequency discontinuous signals and low-frequency ratio signals. What is more, this method requires less computation time, which is very important with respect to real-time signal processing.
(4) In order to verify the effectiveness of the revised HHT, the original HHT and revised HHT are applied to identify the running status of a rotor system, respectively. In the experiment, the vibration displacement signals of a rotor system under normal, rubbing, and misalignment conditions were measured by eddy current sensors. Then, the signals were analyzed by the original and revised HHT methods. The experimental results illustrate that both of the HHT methods can identify the three different running states according to the time-frequency spectrum. By comparing the two methods, we can come to the conclusion that the revised HHT is more reliable than the original with respect to eliminating end effects and mode mixing.
(5) It is worth noting that, although the revised HHT method provides us with good performance in eliminating end effects and mode mixing, it still depends on experience to decide the frequency and amplitude of the added high-frequency sinusoidal signal. More theoretical analysis is necessary if we want to improve the accuracy of the revised HHT. On the positive side, this article provides a new way to improve the performance of HHT.

## Figures and Tables

**Table 1:** The root mean square error (RMSE) values.

	RMSE
The proposed method	0.3057
Yang’s method [28]	0.6548

**Table 2:** The computation time consumption of each method.

Item	Time (s)					
Label	A	B	C	D	E	Average
The revised EMD	0.53	0.57	0.53	0.58	0.55	0.55
EEMD	118.65	118.23	118.03	117.40	118.01	118.06

**Table 3:** The correlation coefficients between each IMF and the original signal.

IMFs	IMF1	IMF2	IMF3	IMF4	r4
Correlation coefficient	0.765	0.638	0.049	0.035	0.024

**Table 4:** The computation time consumption of each method.

Item	Time (s)					
Label	A	B	C	D	E	Average
The proposed method	0.93	0.98	0.95	0.95	0.95	0.95
The original method	1.28	1.29	1.28	1.30	1.30	1.29
